# Prevalence of human papillomavirus in eyelid carcinoma among Koreans: a clinicopathological study

**DOI:** 10.1186/s12886-023-03131-9

**Published:** 2023-09-26

**Authors:** Min Kyu Yang, Namju Kim, Hokyung Choung, Ji Eun Kim, Sang In Khwarg

**Affiliations:** 1 Department of Ophthalmology, Asan Medical Center, Seoul, Korea; 2 Department of Ophthalmology, Seoul National University Bundang Hospital, Seongnam, Korea; 3 Department of Ophthalmology, Seoul Metropolitan Government–Seoul National University Boramae Medical Center, 20 Boramae-ro 5-gil, Dongjak-gu, 07061 Seoul, Republic of Korea; 4 Department of Pathology, Seoul Metropolitan Government–Seoul National University Boramae Medical Center, 20 Boramae-ro 5-gil, Dongjak-gu, 07061 Seoul, Republic of Korea; 5 Department of Ophthalmology, Seoul National University Hospital, Seoul, Korea

**Keywords:** DNA microarray, Eyelid, Human papillomavirus, Sebaceous gland carcinoma, Squamous cell carcinoma

## Abstract

**Background:**

Human papillomavirus (HPV) has been detected in eyelid sebaceous gland carcinoma (SGC) and squamous cell carcinoma (SCC), and detection rates greatly varied across studies. This study aimed to investigate the presence of HPV in eyelid SGC and SCC among Koreans and its correlation with clinicopathological characteristics.

**Methods:**

Surgically resected eyelid samples diagnosed as SGC or SCC from January 1999 to June 2011 were identified from the pathology database of three referral centres in Korea. Clinicopathological information including origin (skin vs. tarsal conjunctiva) and treatment outcomes were retrospectively reviewed. Immunohistochemistry (IHC) for p16, HPV DNA in situ hybridisation (ISH), and polymerase chain reaction-based DNA microarray were performed in paraffin-embedded tissue sections.

**Results:**

Our cohort included 34 SGC and 12 SCC cases with Asian ethnicity. HPV was detected in 4 SGC and 6 SCC by DNA microarray, while 2 SCC (16.7%) showed positivity in ISH. SCC of tarsal conjunctival origin was significantly more common in HPV-positive SCC than in HPV-negative SCC (5 of 6 vs. 0 of 6, *P* = 0.015, Fisher’s exact test). Among samples showing positive staining in p16 IHC, HPV positivity rates were 0.0% (0/19) in SGC and 100% (3/3) in SCC. There was no significant difference in overall and local recurrence rate in eyelid SGC and SCC according to the HPV status (*P* > 0.99).

**Conclusions:**

HPV was found in a subset of eyelid SGC and SCC among Koreans and might be aetiologically related to SCC of tarsal conjunctival origin. Overexpression of p16 is considered to be inappropriate as an indicator of HPV infection in eyelid SGC. Further investigation is required to elucidate the transmission route and pathogenic roles of HPV.

## Background

Among non-melanocytic skin cancers, the most common are basal cell carcinoma and squamous cell carcinoma (SCC) [1]. However, due to the rich distribution of sebaceous glands in the eyelids, such as the Meibomian glands, the incidence of sebaceous gland carcinoma (SGC) is significantly higher than that of other organs [2, 3]. The proportions of SGC and SCC were relatively higher in East Asia than in the other areas [1, 4–6]. and the incidence of eyelid SGC has gradually increased in Korea [7].

Since the eyelids are covered with thin skin and mucosa, external stimuli have been pointed out as the causative factors of eyelid cancers [2]. Microorganisms are also thought to contribute to carcinogenesis in the skin and mucosa [8]. Particularly, human papillomavirus (HPV) is recognised as the direct cause of SCC in the anogenital tract or the oropharynx [9]. Also, HPV has been found in ocular surface squamous neoplasm (OSSN) in several studies [10–12]. However, these studies for SCC were performed using limited samples or yielded inconsistent data [13–15]. For eyelid SGC, HPV detection rates greatly varied across studies [16–23]. Tetzlaff et al. [17] reported exclusively higher prevalence of HPV in tumors wild type for TP53 and RB1. Inactivation of tumor suppressor proteins TP53 and RB by expression of viral proteins E6 and E7 has been proposed as a oncogenic mechanism in OSSN and eyelid SGC [14, 17].

In this study, we investigated the presence of HPV in eyelid SGC and SCC in Koreans and its correlation with clinicopathological characteristics.

## Methods

### Case selection

Surgically resected eyelid samples diagnosed as SGC or SCC at three referral centres in Korea (Seoul National University Hospital, Seoul National University Bundang Hospital, Seoul Metropolitan Government-Seoul National University Boramae Medical Center) from January 1999 to June 2011 were identified via a retrospective review of the pathology database and included in this study. Clinicopathological information including patient demographics, origin (cutaneous vs. tarsal conjunctiva) and depth of invasion, specific pathologic features, recurrence, and metastasis were obtained from electronic medical records. Exclusion criteria were poorly differentiated carcinoma and the presence of ocular surface lesions (e.g. pterygium). TNM staging was estimated according to the 8th edition of the American Joint Committee on Cancer staging system [24]. This study was approved by the Institutional Review Board of the Seoul Metropolitan Government-Seoul National University Boramae Medical Center. This study was conducted in compliance with the Declaration of Helsinki.

### Tissue microarray

The formalin-fixed, paraffin-embedded (FFPE) tissue blocks were retrieved from the pathology archives. The hematoxylin and eosin-stained slides were reviewed to confirm the diagnosis, and the representative area was marked by a pathologist (JEK). From every block, a cylinder of 2.0-mm-diameter was taken and transferred to the recipient paraffin block that contained 60 tissue cores. The 5-µm-thick sections were used for the p16 immunohistochemistry (IHC) and DNA in situ hybridisation (ISH).

### p16 IHC

After deparaffinisation and rehydration, heat-induced antigen retrieval was performed by microwave oven for 20 min in a citrate buffer. Endogenous peroxidase was blocked by 0.3% hydrogen peroxide in methanol for 10 min, and then nonspecific antibody binding was blocked by 10% normal goat serum. IHC was performed using the Benchmark Ultra automated immunostainer (Ventana Medical Systems, AZ, USA). Immunoreactivity against anti-p16 mouse monoclonal antibody (clone JC8, Santa Cruz Biotechnology, Inc., CA, USA) was visualised using the iVIEW detection kit (Ventana Medical Systems) containing the biotin-conjugated secondary antibody, streptavidin-hydrogen peroxide conjugate, and diaminobenzidine. The p16 IHC was interpreted as ‘positive’ when the percentage of stained nuclear and cytoplasmic cells was > 70% of cancer cells. Patchy or focal expression in the surface epithelial cells was regarded as negative.

### DNA ISH

DNA ISH was performed using the Benchmark Ultra automated immunostainer (Ventana Medical Systems) according to the manufacturer’s instructions. Inform HPV III probe sets (Ventana Medical Systems) can identify 12 types of mucosal high-risk (16, 18, 31, 33, 35, 39, 45, 51, 52, 56, 58, and 66) and low-risk (6, 11) HPV [25]. Appropriate positive controls consisting of HPV-related cervical SCC were also applied. Any number of dot-like or scattered tiny staining (punctate pattern) and a globular homogeneously dense staining (diffuse pattern) localised in the tumour cell nuclei were interpreted as positive by microscopic examination [26].

### DNA extraction

Microtome blades were replaced between samples, and the sterility of the machine was maintained to prevent cross-contamination of HPV DNA [27]. DNA was isolated using a QIAamp DNA FFPE tissue kit (Qiagen Inc., Hilden, Germany). Briefly, the paraffin was dissolved in xylene, followed by centrifugation to remove condensate. The tissue sample was then incubated with 20 µL proteinase K at 56 ℃ until completely lysed. The lysed emulsion was further purified using a QIAamp MinElute column (Qiagen Inc.) and centrifugation. DNA was finally eluted by adding 100 µL ATE buffer.

### DNA microarray

DNA microarray, which is highly sensitive and specific for the detection and genotyping of HPV [20, 28, 29], was used. PCR amplifications were performed using consensus primers for E6/E7 and L1 gene sequences of HPV (HPCF, HPCR, MY11, GP6-1) and human beta-globin gene (HBBF, HBBR) [30]. PCR-based DNA microarray was performed using the GG HPV DNA Genotyping Chip Kit (Goodgene, Seoul, Korea). The GG DNA Chip contains 41 mucosal type-specific probes that recognise 22 high-risk (16, 18, 26, 31, 33, 35, 39, 45, 51, 52, 53, 56, 58, 59, 66, 67, 68a, 68b, 69, 70, 73, and 82) and 19 low-risk (6, 11, 30, 32, 34, 40, 42, 43, 44, 54, 55, 61, 62, 72, 81, 83, 84, 90, and 91) HPV types [31]. According to the manufacturer’s instructions, 10 µL of PCR-amplified HPV DNA and human beta-globin gene product were denatured by heating at 95 °C for 3 min, followed by ice-cooling for 3 min. The amplified product was mixed with 65 µL hybridisation buffer and then applied to the DNA microarray. Hybridisation was performed at 45 °C for 1 h, followed by washing with 3X saline-sodium phosphate-ethylenediaminetetraacetic acid for 3 min and drying at room temperature. The hybridisation signal was scanned using an Affymetrix 428 Array Scanner (Affymetrix Inc., CA, USA).

### Statistical analysis

Statistical analyses were performed using IBM SPSS Statistics software (version 22.0, IBM Corp., NY, USA). Mann–Whitney U test was used for the comparison of continuous variables, and Fisher’s exact test for categorical variables. A two-sided *P*-value < 0.05 was considered to be statistically significant.

## Results

### Patients’ characteristics

Our cohort included 34 SGC and 12 SCC patients with a mean age of 61.4 ± 13.3 years (range, 26.8 to 88.6 years) of Asian ethnicity (Table 1). All the patients were seronegative for human immunodeficiency virus, and immunocompetent. All SCC did not involve superior fornix and bulbar conjunctiva, thus could be distinguished from OSSN. Surgical excision with frozen section control was performed primarily in all patients. Radiotherapy was applied for one SGC and one SCC with orbital invasion. Ten SGC patients exhibited later recurrence (local recurrence: 3, lymph node metastasis: 6, lung metastasis: 1) at an average of 1.7 ± 1.0 years (range, 0.6–3.1 years). Locally recurred three SGC were treated by exenterations. An SCC patient showed a postoperative 3-year recurrence at the ipsilateral preauricular lymph node and an 8-year local recurrence.

**Table 1:** Clinicopathological Characteristics of 46 Patients with Eyelid Carcinoma

n = 46	SGC (n = 34)	SCC (n = 12)
Male sex, n (%)	9 (26.5)	6 (50.0)
Age at surgery, median (IQR), years	60.6 (51.8–65.8)	65.0 (55.5–74.5)
Location, n (%)		
Upper eyelid	18 (52.9)	6 (50.0)
Lower eyelid	15 (44.1)	6 (50.0)
Upper and lower eyelid	1 (2.9)	0 (0.0)
Depth of invasion, n (%)		
Skin only	0 (0.0)	5 (41.7)
Tarsal plate or lid margin	15 (44.1)	5 (41.7)
Full thickness of the eyelid	19 (55.9)	2 (16.7)
Periorbital or orbital invasion, n (%)		
Lacrimal drainage system	1 (2.9)	1 (8.3)
Orbit	3 (8.8)	2 (16.7)
T category at presentation, n (%)		
T1	18 (52.9)	9 (75.0)
T2	10 (29.4)	1 (8.3)
T3	1 (2.9)	0 (0.0)
T4	5 (14.7)	2 (16.7)
N category at presentation, n (%)		
N1	2 (5.9)	0 (0.0)
Specific histology, n (%)		
Pagetoid spread	3 (8.8)	-
Keratoacanthoma-type	-	1 (8.3)
Primary surgical procedure, n (%)		
Wide excision with reconstruction	31 (91.2)	12 (100.0)
Exenteration	3 (8.8)	0 (0.0)
Treatment outcomes, n (%)		
Local recurrence	3 (8.8)	1 (8.3)*
Lymph node metastasis	6 (17.6)	1 (8.3)*
Distant metastasis	1 (2.9)	0 (0.0)

*IQR* interquartile range, *SCC* squamous cell carcinoma, *SGC* sebaceous gland carcinoma

*Postoperative 3-year metastasis at ipsilateral preauricular lymph node and 8-year local recurrence in a patient

### DNA microarray and DNA ISH

Both tests were performed for all samples. DNA microarray detected HPV DNA in four SGC and six SCC (positivity rates: 11.8% and 50.0%, respectively). The results of HPV detection are listed in Table 2. All samples harboured high-risk HPV, with type 16 being the most common (50.0% of HPV-positive SGC and 66.7% of HPV-positive SCC).

**Table 2:** Results of Human Papillomavirus (HPV) Detection Methods in 10 Eyelid Carcinomas with HPV Positivity in DNA Microarray

No.	Sex	Age	Histology	Genotypes		HPV ISH	p16 IHC
				High-risk	Low-risk		
1	F	41–50	SGC	16	Negative	Negative	Negative
2	M	61–70	SGC	16	43	Negative	Negative
3	M	31–40	SGC	18	Negative	Negative	Not available
4	F	61–70	SGC	56	Negative	Negative	Not available
5	M	61–70	SCC	16, 66	Negative	Diffusely positive	Positive
6	M	81–90	SCC	16	Negative	Diffusely positive	Positive
7	M	51–60	SCC	51	Negative	Negative	Positive
8	M	51–60	SCC	16	Negative	Negative	Negative
9	F	71–80	SCC	16	Negative	Negative	Negative
10	F	71–80	SCC	68b	Negative	Negative	Negative

*IHC* immunohistochemistry, *ISH* in situ hybridization, *SCC* squamous cell carcinoma, *SGC* sebaceous gland carcinoma

**Fig. 1:**
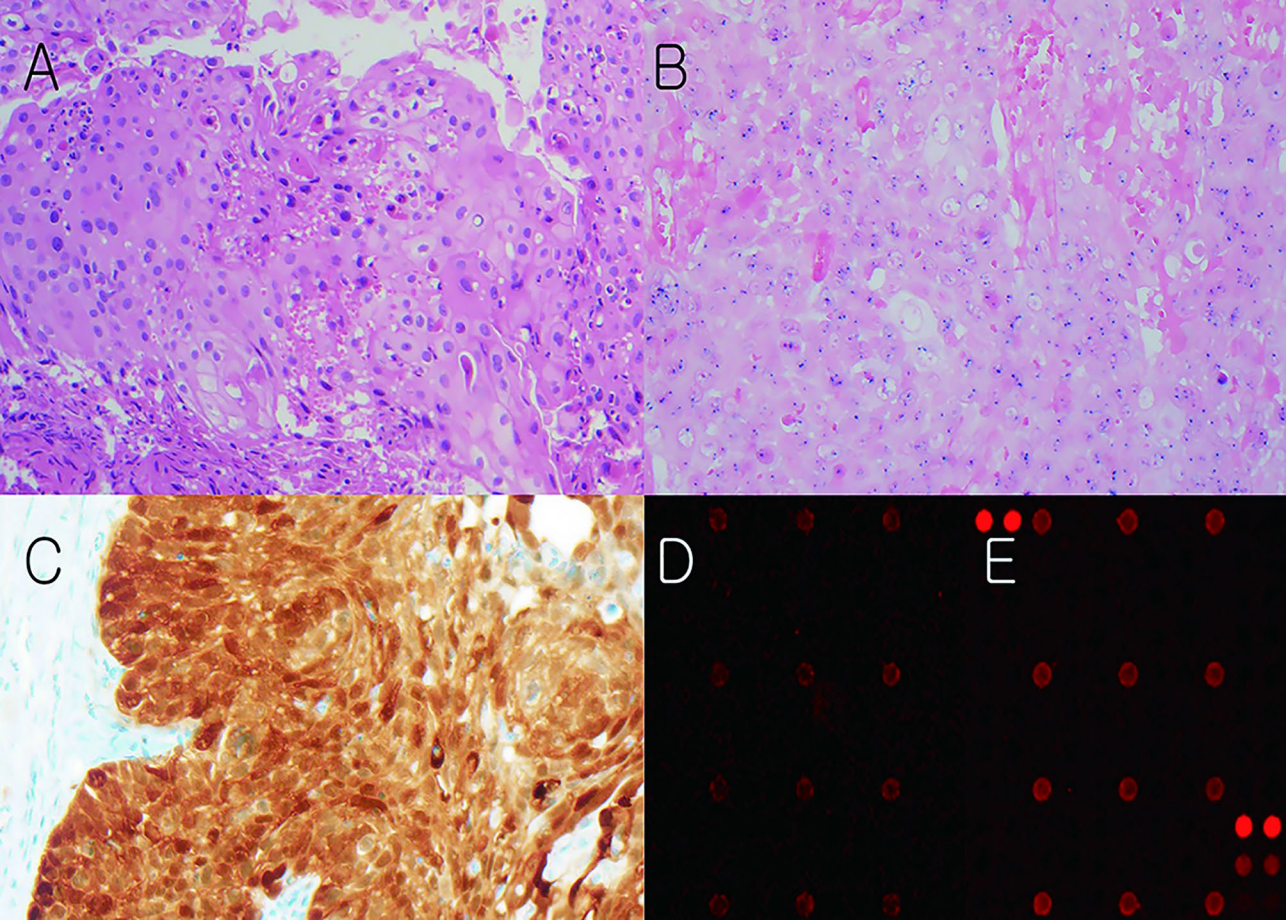
A representative case of eyelid squamous cell carcinoma with human papillomavirus (HPV). **A**: Hematoxylin and eosin staining. **B**: In situ hybridization shows a positive for HPV DNA. **C**: p16 immunohistochemistry shows positive staining. **D**: DNA microarray shows signals of HPV type 16 and **E**: HPV type 66

By DNA ISH, HPV was not detected in SGC, while two cases of SCC (16.7%) showed diffuse strong signals. These cases presented as multiple lesions in the tarsal conjunctiva, showed positive staining in p16 IHC, and were genotyped as HPV type 16 by DNA microarray (Fig. 1). Most patients presenting HPV positivity in DNA microarray but not in DNA ISH showed negative staining in p16 IHC.

### p16 IHC

Among 30 SGC evaluated for p16 IHC, 19 SGC (63.3%) showed positive, and 11 SGC showed negative staining as per the criteria mentioned above. Among nine SCC evaluated for p16 IHC, only three SCC (33.3%) showed positive staining in p16 IHC. Among samples showing positive staining in p16 IHC, HPV-positive rates were 0.0% (0/19) in SGC and 100% (3/3) in SCC.

### Association of HPV positivity with clinicopathological characteristics

SGC and SCC showed different characteristics in terms of HPV positivity in DNA microarray (Table 3). HPV-positive SGC patients were slightly younger than HPV-negative SGC patients (54.4 years vs. 60.4 years, *P* = 0.18, Mann–Whitney U test). Distributions of the depth of invasion and T-staging were not different according to the HPV positivity in SGC. HPV-positive SGC showed lower overall and local recurrence rates than HPV-negative SGC (overall recurrence rate: 0.0% vs. 30.0%, *P* = 0.55, Fisher’s exact test).

The origin and depth of invasion of eyelid SCC were different according to HPV positivity. SCC of tarsal conjunctival origin, whose size was largest at the tarsal conjunctiva in slit-lamp examination and confirmed by pathologic review, was significantly more common in HPV-positive SCC than in HPV-negative SCC (5 of 6 vs. 0 of 6, *P* = 0.02, Fisher’s exact test). Conversely, all SCC of tarsal conjunctival origin were HPV-positive (5 of 5). There was no significant difference in recurrence rates according to the HPV status (1 of 6 HPV-positive SCC vs. 0 of 6 HPV-negative SCC, *P* > 0.99).

**Table 3:** Clinicopathological Characteristics of Eyelid Carcinoma According to Human Papillomavirus (HPV) Positivity in DNA Microarray

	SGC		*p* value	SCC		*P* value
	HPV (+) (n = 4)	HPV (-) (n = 30)		HPV (+) (n = 6)	HPV (-) (n = 6)	
Male sex, n (%)	2 (50.0)	7 (23.3)	0.28	2 (33.3)	4 (66.7)	0.57
Age at surgery, median (IQR), years	54.4 (42.7–61.6)	60.4 (52.2–68.1)	0.18	71.5 (63.3–78.1)	64.0 (54.9–66.0)	0.20
Upper eyelid location, n (%)	1 (25.0)	17 (56.7)	0.32	4 (66.7)	2 (33.3)	0.57
Depth of invasion, n (%)						
Skin only	0 (0.0)	0 (0.0)	> 0.99	1 (16.7)	4 (66.7)	0.24
Tarsal plate*	4 (100.0)	30 (100.0)	> 0.99	5 (83.3)	1 (16.7)	0.08
Origin, n (%)†						
Tarsal conjunctival origin	4 (100.0)	28 (93.3)	> 0.99	5 (83.3)	0 (0.0)	**0.02**
Cutaneous origin	0 (0.0)	0 (0.0)	> 0.99	1 (16.7)	6 (100.0)	**0.02**
Orbit invasion, n (%)	0 (0.0)	3 (10.0)	> 0.99	2 (33.3)	0 (0.0)	0.46
T category at presentation, n (%)						
T1	2 (50.0)	16 (53.5)	0.66	6 (100.0)	3 (50.0)	0.18
T1 or T2	4 (100.0)	24 (80.0)	> 0.99	6 (100.0)	4 (66.7)	0.46
Pagetoid spread, n (%)	0 (0.0)	3 (10.0)	> 0.99	0 (0.0)	0 (0.0)	> 0.99
Treatment outcome, n (%)						
Overall recurrence	0 (0.0)	9 (30.0)	0.55	1 (16.7)‡	0 (0.0)	> 0.99
Local recurrence	0 (0.0)	3 (10.0)	> 0.99	1 (16.7)‡	0 (0.0)	> 0.99

*IQR* interquartile range, *SCC* squamous cell carcinoma, *SGC* sebaceous gland carcinoma

Bold indicates statistical significance

*Including tarsal plate involvement and full-thickness involvement

†Largest lesion size at the level of tarsal conjunctiva or skin

‡Postoperative 3-year metastasis at ipsilateral preauricular lymph node and 8-year local recurrence in a patient

## Discussion

This study investigated the association of HPV in eyelid SGC and SCC with clinicopathological characteristics. Prevalence of HPV was higher in SCC than in SGC, being particularly more frequent in SCC of the tarsal conjunctival origin. DNA microarray detected the HPV genome more sensitively than DNA ISH.

The pathogenesis of SGC and SCC of the eyelid is largely unknown, however, the role of *TP53* or *RB1* gene alteration or dysregulation has been consistently suggested [32–35]. Inactivation of these two molecules, a common event for carcinogenesis, can be a consequence of interaction with HPV oncoprotein which also triggers oxidative stress-induced DNA damage [36, 37]. Several researchers have investigated whether HPV is involved in the pathogenesis of eyelid SGC, and the results reported so far vary widely. In 1994, Hayashi et al. [21] investigated p53 protein accumulation in eyelid SGC and reported a high frequency of HPV DNA. However, subsequent studies showed low rates of HPV (0 ~ 4.2%) and failed to prove an association between eyelid SGC and HPV [16, 18–20, 22, 23]. The inconsistent results of the previous studies are thought to be primarily due to differences in testing sensitivity and may additionally include racial or regional differences and increasing frequency. The detection rate of HPV in our SGC samples based on DNA microarray was similar to that of a most recent study in the USA (13.8%) using whole transcriptome RNA sequencing and RNA ISH [17]. Further investigation of the p53/Rb protein and transcriptionally active HPV in Asians will be helpful to clarify the role of HPV in the pathogenesis of eyelid SGC.

In our study, the clinical characteristics of eyelid SGC and SCC differed according to the association with HPV. Although the differences were not statistically significant, patients with HPV-positive SGC were younger and had a lower recurrence rate, which is consistent with previously reported findings [17]. In eyelid SCC, these trends were not observed, rather the tarsal conjunctival origin was associated with HPV positivity. The eyelid is a complex of various tissues, and its external and internal surfaces are covered with two different types of epithelium: skin and conjunctiva. These differ in histological characteristics and exposure to oncogenic stimuli. Unlike the skin, the tarsal conjunctiva has no exposure to UV radiation and has a histological resemblance with the bulbar conjunctiva. Therefore, it is not unusual to infer that eyelid SCC with tarsal conjunctival origin has an aetiology similar to OSSN rather than eyelid SCC with skin origin. Although still debatable, HPV has been suggested as an aetiologic factor of OSSN [10–12], especially in non-African countries [38]. Our results possibly indicate that HPV serves as an aetiologic factor only for eyelid SCC of tarsal conjunctival origin, and support the association between OSSN and HPV. However, since the route or prevalence of HPV infection in tarsal conjunctiva is not well known, it is difficult to discuss the clinical benefits of HPV screening or vaccination [39].

The diagnostic accuracy of HPV is variable depending on the detection method and tissue type. Numerous PCR techniques have been widely used due to their excellent diagnostic accuracy but also have some drawbacks in detecting multiple infections or genotyping [40]. DNA microarray is highly sensitive and is advantageous for overcoming the drawbacks of conventional PCR [30, 40]. DNA microarray in our study had the broader detection range of HPV types, and detected the HPV genome more sensitively than DNA ISH. ISH has the advantage of morphologic correlation but also has difficulty with low sensitivity if the copy number is low or when HPV is in a dormant period [41]. HPV positivity on microarray but not on ISH indicates latent infection of HPV [42], or false-positivity due to contamination or cross-reactivity [27, 40]. Therefore, multiple detection methods should be applied, and the fact that p16 was mostly negative in such SCC cases supports the former hypothesis. Two cases revealed multiple infection, containing HPV 16 and other types. However, the pathologic significance of HPV depends on the type (e.g. HPV 16, the most prevalent and oncogenic high-risk HPV) but not on the multiplicity or viral load [43]. Thus, other types of co-infected HPV might be ‘bystanders’ that are not primarily involved in the pathogenesis.

Although p16 is known to be associated with HPV infection in anogenital SCC and OSSN, its value is not very strongly recognised in eyelid cancers. In our study, HPV was detected in all eyelid SCC of tarsal conjunctival origin, but not in all eyelid SGC. Mutation of *TP53* or *RB1* is the most frequently encountered alteration in SGC, subsequently resulting in p16 overexpression regardless of HPV infection [44]. Therefore, SGC cases showing p16 positivity without HPV might harbour these molecular alterations.

The route of transmission of HPV in ocular cancer can be postulated in various ways. The conjunctival mucosa, the lacrimal sac, and the hair bulb including follicular cells are supposed reservoirs of HPV [45, 46]. Given that most HPVs are transmitted through sexual contact, it is difficult to infer the pathways of HPV-related tumours in non-sexual organs. Some researchers have suggested intrafamilial spread or vertical transmission through an infected birth canal [47].

Our study has some limitations, including its retrospective design. Although this is the largest study in Asia to our knowledge, the number of samples was small. A larger study using HPV mRNA-based assays and RNA sequencing is required to verify the pathogenic role of transcriptionally active mucosal HPV for the development of eyelid SCC of tarsal conjunctival origin.

In conclusion, HPV was found in a subset of eyelid SGC and SCC among Koreans, and might be aetiologically related to SCC of tarsal conjunctival origin. Overexpression of p16 is considered to be inappropriate as an indicator of HPV infection in eyelid SGC. Further investigation is required to elucidate the transmission route and pathogenic roles of HPV.

## Data Availability

The datasets used and/or analysed during the current study are available from the corresponding author on reasonable request.
